# Common variant of *ALPK1* is not associated with gout: a replication study

**DOI:** 10.1007/s13577-014-0103-1

**Published:** 2014-10-19

**Authors:** Toshinori Chiba, Hirotaka Matsuo, Masayuki Sakiyama, Akiyoshi Nakayama, Seiko Shimizu, Kenji Wakai, Shino Suma, Hiroshi Nakashima, Yutaka Sakurai, Toru Shimizu, Kimiyoshi Ichida, Nariyoshi Shinomiya

**Affiliations:** 1 Department of Integrative Physiology and Bio-Nano Medicine, National Defense Medical College, Namiki 3-2, Tokorozawa, Saitama 359-8513 Japan; 2 Department of Preventive Medicine, Nagoya University Graduate School of Medicine, Nagoya, Japan; 3 Department of Preventive Medicine and Public Health, National Defense Medical College, Tokorozawa, Japan; 4 Midorigaoka Hospital, Takatsuki, Japan; 5 Kyoto Industrial Health Association, Kyoto, Japan; 6 Department of Pathophysiology, Tokyo University of Pharmacy and Life Sciences, Tokyo, Japan

**Keywords:** Gouty arthritis, Uric acid, Urate, ABCG2/BCRP, Gout-susceptibility locus

## Abstract

Gout is one of the most kinds of common inflammatory arthritis as a consequence of hyperuricemia. Alpha-protein kinase 1 (*ALPK1*) gene locates in a gout-susceptibility locus on chromosome 4q21–31, and encodes ALPK1 protein which plays a pivotal role in the phosphorylation of myosin 1. In the previous genetic study of Taiwanese populations, 3 single nucleotide polymorphisms (SNPs), rs11726117, rs231247 and rs231253, in *ALPK1* gene were reported to have a significant association with gout. However, no replication study has been performed to confirm this association. Therefore, we first conducted a replication study with clinically defined gout patients in a different population. Linkage disequilibrium (LD) analyzes of the 3 SNPs in *ALPK1* revealed that these SNPs are in strong LD in a Japanese population. Among the 3 SNPs of *ALPK1*, rs11726117 (M861T) is the only missense SNP. Therefore, rs11726117 was genotyped in a Japanese population of 903 clinically defined gout cases and 1,302 controls, and was evaluated for a possible association with gout. The minor allele frequencies of rs11726117 were 0.26 and 0.25 in the case and control groups, respectively. The association analysis has not detected a significant association between rs11726117 and gout susceptibility in a Japanese population (*p* = 0.44). Because *ABCG2*, a major causative gene for gout, also locates in the gout-susceptibility locus on chromosome 4q, these findings suggest that among genes in a gout-susceptibility locus, not *ALPK1* but *ABCG2* could be important as a gout-susceptible gene.

## Introduction

Gout, a multifactorial disease, is characterized by acute inflammatory arthritis which induces severe painful attacks. Gout is caused as a consequence of hyperuricemia. Previous genetic studies have revealed that gout has associations with various genes such as ATP-binding cassette transporter, subfamily G, member 2 (*ABCG2/BCRP*) [1–4], monocarboxylate transporter 9 (*MCT9/SLC16A9*) [5], organic anion transporter 4 (*OAT4/SLC22A11*) [6], leucine-rich repeat-containing 16 A (*LRRC16A/CARMIL*) [7], and alpha-protein kinase 1 (*ALPK1*) [8].

ALPK1 is thought to play a pivotal role in the phosphorylation of myosin 1 and the apical trafficking of raft-associated sucrose–isomaltase [9]. In the previous study of Taiwanese Han and Taiwan aborigines, Ko et al. [8] reported that 3 single nucleotide polymorphisms (SNPs), rs11726117, rs231247 and rs231253, in *ALPK1* gene are associated with gout. However, no replication study has been performed to confirm the association between *ALPK1* and gout.

In the present study, we therefore investigated the association between gout and *ALPK1* with Japanese gout cases and controls.

## Materials and methods

### Study participants

As cases, 903 male Japanese patients with primary gout were collected from the outpatients of Midorigaoka Hospital (Osaka, Japan), Kyoto Industrial Health Association (Kyoto, Japan) and Jikei University Hospital (Tokyo, Japan). Gout diagnoses were obtained according to the criteria established by the American College of Rheumatology [10]. For controls, 1,302 male Japanese individuals were collected from the Japan Multi-Institutional Collaborative Cohort Study (J-MICC Study) [11]. Exclusion criteria for the controls were high serum uric acid (SUA) levels (>7.0 mg/dl) and any gout history. The mean ages with standard deviation of case and control groups were 55.2 ± 12.9 and 52.7 ± 8.4 years old, respectively, and their respective mean body-mass index was 24.7 ± 3.3 and 23.2 ± 2.8 kg/m^2^. In this study, all subjects provided written informed consent. This study was approved by the institutional ethical committees, and all procedures involved in this study were performed in accordance with the Declaration of Helsinki.

### Linkage disequilibrium analysis

Using the Phase III HapMap JPT (Japanese in Tokyo) data [12], linkage disequilibrium analyzes have been performed among rs11726117, rs231247 and rs231253 with software R (version 3.1.0) (http://www.r-project.org/) with package GenABEL.

### Genotyping

Genomic DNA was extracted from whole peripheral blood cells [13]. Genotyping of rs11726117 was performed by the TaqMan method (Life Technologies Corporation, Carlsbad, CA, USA) with a LightCycler 480 (Roche Diagnostics, Mannheim, Germany) [14, 15]. To confirm their genotypes, more than 30 samples were subjected to direct sequencing with the following primers: forward 5′-ACCCTTCTGGCCTCATAATAC-3′, and reverse 5′-CTTTACAACCATTAAGGTCCATC-3′. DNA sequencing analysis was performed with a 3130xl Genetic Analyzer (Life Technologies Corporation) [15].

The *χ* ^2^ test was used for association analysis with SPSS v.22.0J (IBM Japan Inc., Tokyo, Japan).

## Results

In the previous genetic analysis of the Taiwanese populations by Ko et al. [8], the genotype distributions are very similar among the 3 SNPs (rs11726117, rs231247 and rs231253) of *ALPK1* (Table 1). Therefore, we hypothesized that these SNPs are in linkage disequilibrium. To confirm this hypothesis, the HapMap JPT data have been analyzed. According to the hypothesis, the 3 SNPs were in strong linkage disequilibrium (*r* ^2^ ≥ 0.99; Table 1) among the Japanese population in HapMap data, indicating that minor alleles of the 3 SNPs exist in one haplotype. Therefore, in this study, rs11726117 was genotyped to reveal its association with gout, because only this SNP is the nonsynonymous mutation (M861T) among these SNPs; rs231247 is a synonymous mutation (R1084R) and rs231253 is in the 3′ untranslated region (3′ UTR).

**Table 1:** Minor allele frequencies and linkage disequilibrium of 3 SNPs of *ALPK1* gene

	A1^a^	A2^a^	Taiwanese Han^b^				Taiwan aborigines^b^				HapMap JPT^c^					
			A1/A1	A1/A2	A2/A2	MAF	A1/A1	A1/A2	A2/A2	MAF	A1/A1	A1/A2	A2/A2	MAF	*r* ^*2* d^	*D*′^d^
rs231247	G	A	204	167	36	0.29	225	414	201	0.49	59	46	8	0.27	0.99	1
rs231253	G	C	215	164	28	0.27	223	416	201	0.49	59	46	8	0.27	0.99	1
rs11726117	C	T	209	168	30	0.28	244	396	200	0.47	57	47	8	0.28	–	–

*MAF* Minor allele frequency

^a^The major allele was referred to A1 and the minor allele as A2

^b^Data from reference 8

^c^Data from the Phase III HapMap JPT (Japanese in Tokyo)

^d^Results of linkage disequilibrium analysis between rs11726117 and rs231247, or between rs11726117 and rs231253

Table 2 shows the genotyping result of rs11726117 for 903 gout patients and 1,302 controls. The call rate was 97.4 %. The frequencies of genotypes were in Hardy–Weinberg equilibrium (*p* = 0.43).

**Table 2:** Association analysis of rs11726117 of *ALPK1* gene in gout cases and controls

	Genotype				Allele frequency mode			
	C/C	C/T	T/T	*p* value	MAF	*p* value	OR	95 % CI
Case	487	338	66	0.75	0.26	0.44	1.05	0.92–1.21
Control	706	465	86	–	0.25	–	Ref	–

*MAF* minor allele frequency, *OR* odds ratio, *CI* confidence interval, *Ref* reference

As compared with the control group, the genotype distribution of rs11726117 (C/C, C/T or T/T) in the case group was not significantly different (*p* = 0.75; Table 2).

The minor allele (T) frequencies of the variant were 0.26 and 0.25 in case and control groups, respectively, indicating that rs11726117 is a common missense mutation. The association analysis has not detected a significant association between rs11726117 and gout susceptibility in the allele frequency mode (*p* = 0.44; Table 2).

## Discussion

*ALPK1* gene locates in a gout-susceptibility locus (between microsatellite markers 4DS3243 and 4DS1625) on chromosome 4q21–31 [16]. In the Taiwanese populations, *ALPK1* was previously reported to be associated with gout susceptibility [8].

ALPK1 belongs to the alpha-kinase family and plays a role in the phosphorylation of myosin 1 [9]. A recent genome-wide association study (GWAS) revealed the possible relationship between *ALPK1* SNPs and chronic kidney disease (CKD) [17]. As hyperuricemia is highly correlated with CKD risk [18, 19], together with the renal expression of ALPK1 [17], *ALPK1* could be a possible susceptible gene for gout/hyperuricemia.

**Fig. 1:**
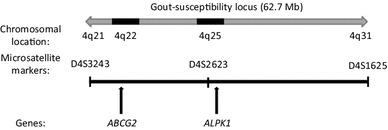
The locations of *ALPK1* and *ABCG2* in the gout-susceptibility locus. Gout-susceptibility locus was previously identified between D4S3243 and D4S1625 on chromosome 4q21–31. Both *ALPK1* and *ABCG2* locate in this locus

However, the present study detected no significant association between *ALPK1* and gout. This may be partly due to the difference of the investigated population. In addition, we previously reported that *ABCG2*, which also locates in a gout-susceptibility locus on chromosome 4q21–31 (Fig. 1), is strongly associated with gout [2, 20]. Taken together, these findings suggest that among genes in a gout-susceptibility locus, not *ALPK1* but *ABCG2* is important as a susceptible gene for gout (Fig. 1). Although further studies of *ALPK1* are necessary to reveal the relationship between *ALPK1* SNPs and gout, our study at least revealed that rs11726117 of *ALPK1* is not a strong genetic risk for gout.
